# Supplementary material for: A set of multi-entry identification keys to African frugivorous flies (Diptera, Tephritidae)
Source: Zookeys. 2014 Jul 24;(428):97–108. doi: 10.3897/zookeys.428.7366 (PMC4143993; doi:10.3897/zookeys.428.7366)
Supplement: Supplementary material 6 — Key to Ceratitis [file zookeys-428-097-s006.zip › SF6_ZooKeys_key to Ceratitis/key/SF6_ZooKeys_key to Ceratitis/Media/Html/Ceratitis bicincta .htm]

Ceratitis (Pterandrus) bicincta Enderlein


***Ceratitis*** **(*Pterandrus*) *bicincta* Enderlein**

*Ceratitis* *bicincta* Enderlein, 1920: 349.

Body
length: 4.06 (3.75-4.40) mm; wing length: 4.23 (4.05-4.55) mm.

Male

Head: Antenna yellowish orange. First flagellomere 2-3 times
as long as pedicel. Arista with short to moderately long rays; basoventral rays
shorter and sparser than basodorsal rays. Frons convex; yellow, in middle and
at frontofacial angle with darker patches, the latter extending partly
downwards along medial eye margin; with short scattered setulae distinctly
darker than frons. Frontal setae well developed. Face white. Genal seta and
setulae black or dark reddish, latter poorly developed. Occiput with dorsal
0.33 dark.

Thorax:
Postpronotal lobe dark yellow, with black spot. Scutal pattern shiny black or
brown, microtrichose areas silvery with ashgray shine, along sutural line
indistinct dark yellow patches, prescutellar yellow markings separate but with
pale area in between. Scapular setae dark. Scutellum yellowish white, basally
with two merged dark spots, apically with three merged black spots, weakly
incised, extending to basal spots or almost so; rarely almost completely black
except basally near lateral margins with paler spot (as in female).
Anepisternum on ventral half brown; setulae pale, except ventral to
anepisternal seta where few dark setae.

Legs:
Yellow; without any feathering except midfemur along ventral margin with few
longer pale setulae distally, hindfemur at apical 0.33 with longer setulae
ventrally and dorsally, but never distinct feathering. Pilosity mainly pale;
forefemur posterior row with dark setulae basally, ventral spines mix pale and
dark.

Wing:
Bands brown. Marginal band forming continuous band with anterior part of discal
band; cubital band free; medial band absent; crossvein R-M proximal to middle
of discal cell. Apex of vein R_1_ distal to level of crossvein R-M.
Crossvein DM-Cu oblique anterobasally.

Abdomen:
Tergite 1 yellowish with brown patches across posterior margin; tergites 2 and
4 anteriorly brown, with silvery transverse band along posterior half; tergite
3 completely brownish black; tergite 5 yellowish brown in the middle, apices
dark brown. Setation and banding typical for subgenus. dark brown. Male
epandrium in lateral view distinctly broadened; base of lateral surstylus
broadened, posterior lobe of lateral surstylus curved.

Female

As
male except for the following characters. Antenna sometimes more brownish,
especially center of third segment. Frons often with short scattered setulae
largely of same color as frons. Genal seta and setulae black and well
developed. Postpronotal spots usually larger, occupying most of the
postpronotal lobe so that it appears to have largely the same color as scutum.
Scutal pattern darker and sometimes more shiny black, spots strongly reduced;
occasionally with largely black pilosity. Scutellum sometimes largely shiny
black, except dark yellow basal spots near lateral margin. Anepisternal
pilosity more extensively dark ventral to anepisternal seta. Legs yellow except
femora brown, tibiae partly yellowish brown; without any feathering. Pilosity
mixed pale and dark; forefemur with posterior row and ventral spines dark.
Abdominal tergite usually darker than in male. Oviscape shorter than
preabdomen, partly black colored. Aculeus at least 10 times longer than wide;
tip blunt, and with lateral margin straight.

(Description
after De Meyer & Freidberg, 2006)
